# Angiopep-2/IP10-EGFRvIIIscFv modified nanoparticles and CTL synergistically inhibit malignant glioblastoma

**DOI:** 10.1038/s41598-018-30072-x

**Published:** 2018-08-27

**Authors:** Xuan Wang, Zhiyong Xiong, Zhen Liu, Xing Huang, Xiaobing Jiang

**Affiliations:** Department of Neurosurgery, Wuhan Union Hospital, Tongji Medical College, Huazhong University of Science and Technology, Wuhan, 430022 China

## Abstract

Preparation of agents that can successfully traverse the blood-brain-barrier (BBB) is a key challenge in brain cancer therapeutics. In this study, angiopep-2 was used as a brain-targeting peptide for preparing multifunctional Angiopep-2-modified poly nanoparticles, angiopep-2 and IP10-EGFRvIIIscFv fusion protein modified nanoparticles. *In vitro* experiments showed a greater uptake of Angiopep-2 modified nanoparticles, also angiopep-2 and IP10-EGFRvIIIscFv fusion protein modified nanoparticles by bEnd.3 cells versus nanoparticles and nanoparticles modified by IP10-EGFRvIIIscFv. Angiopep-2 and IP10-EGFRvIIIscFv fusion protein modified nanoparticles accumulated in brain tissue after intravenous injection and recruited activated CD8^+^ T lymphocytes to location of glioblastoma cells. *In vivo* experiments to assess anti-glioblastoma effect of angiopep-2 and IP10-EGFRvIIIscFv fusion protein modified nanoparticles showed significantly reduced tumor volume in angiopep-2 and IP10-EGFRvIIIscFv fusion protein modified nanoparticles^+^ CD8^+^ cytotoxic T lymphocytes group versus in NPs modified by IP10-EGFRvIIIscFv^+^ CD8^+^ cytotoxic T lymphocytes, CD8^+^ cytotoxic T lymphocytes, Angiopep-2 modified nanoparticles^+^ CD8^+^ cytotoxic T lymphocytes, angiopep-2 and IP10-EGFRvIIIscFv fusion protein modified nanoparticles and PBS groups. Leukocytes infiltrated in brain tissues showed strong anti-glioblastoma activity in angiopep-2 and IP10-EGFRvIIIscFv fusion protein modified nanoparticles^+^ CD8^+^ cytotoxic T lymphocytes treated mice. Thus, angiopep-2 and IP10-EGFRvIIIscFv fusion protein modified nanoparticles may be useful for brain-targeted delivery and recruitment of activated CD8^+^ T lymphocytes to glioblastoma cells.

## Introduction

Glioblastoma is the most common malignant primary brain tumor with high relapse rates, mortality and poor prognosis^1,2^. Median survival of glioblastoma patients is <15 months even after individualized therapies^3,4^. The standard approach to managing glioblastoma is maximal surgery followed by radiotherapy with adjuvant chemotherapy^5,6^. However, glioblastoma patients are often treatment-resistant and do not achieve much survival benefit^7–9^.

Growing evidences show that tumor-infiltrating lymphocytes are key determinants of therapeutic efficacy of conventional chemotherapy and immunotherapy^10^. Indeed, lymphocyte infiltration in primary brain tumor tissues is a positive prognostic factor in glioblastoma patients. Therefore, immune-targeted therapeutic strategy against glioblastoma has evoked much interest^11^. Immunotherapy targets microscopic tumor and controls metastasis, especially to sites that are unreachable for surgery. Moreover, its low toxicity, high specificity and long-term effects make immunotherapy an ideal adjuvant therapy for targeting residual cancer cellsCarpentier and Meng^12^.

Appropriate tumor rejection antigen conditions can support the production of long lived and functional antitumor specific T cells. Selecting tumor rejection antigen is not a major obstacle as several potential tumor rejection antigens have been identified^13,14^. However, the targeted delivery of antigens is a greater challenge. With exploration of glioblastoma-specific antigens, glioblastoma specific cytotoxic T lymphocytes (CTL) have acquired centerstage in anti-glioblastoma research.

EGFRvIII is amplified in 20–25% of patients with glioblastoma. It represents the most prevalent epidermal growth factor receptor (EGFR) mutation found in human glioblastoma, while it is not readily found in normal brain tissues^15^. The constitutively activated variant EGFRvIII is present in cells with *EGFR* gene amplification and is believed to increase the tumorigenic potential, particularly in glioblastoma cells. The expression of EGFRvIII specifically in glioblastoma cells makes it an appropriate target for glioblastoma immunotherapy^15–17^.

Immune response is controlled by a complex molecular-network, which includes cytokines and chemokines^18^. Chemokines are small secreted proteins that activate several distinct signaling pathways and bring about directed cell migration along the chemokine gradient^19^. Interferon (IFN)-γ-inducible protein (IP) 10, known as CXCL-10, is a CXC chemokine secreted by epithelium, endothelium, keratinocytes, fibroblasts, and monocytes^20,21^. In addition to its strong chemotaxis and functional activation on T cells and B cells, monocytes and macrophages, dendritic cells, NK cells (natural killer cells) and basophils, IP10 is a potent inhibitor of angiogenesis and shows a potent antitumor effect^22,23^. However, IP10 alone may not completely induce tumor regression, which suggests that combination therapies that directly target cancer cells are necessary.

Despite the encouraging results of immunotherapy on glioblastoma *in vitro*, its efficacy against glioblastoma *in vivo* is not ideal. The existence of immune escape mechanisms prevents enough glioblastoma-specific CTLs to accumulate around glioblastoma cells. Therefore, strategies for recruitment of sufficient glioblastoma-specific CTLs around the targeted tumor cells are critical for CTL adoptive treatment of glioblastoma^24,25^.

The engineered therapeutic single-chain Fv (scFv) fragments, EGFRvIIIscFv, have been shown to specifically target EGFRvIII-expressing U87 glioblastoma cells *in vivo*^26^. Therefore, the fusion protein of IP10-EGFRvIIIscFv, which has the potential to specifically bind to EGFRvIII antigen on glioblastoma cells, may enhance the IP10 concentration around the glioblastoma cells and increase CTL infiltration into the glioblastoma.

The existence of a blood brain barrier (BBB) shadows the therapeutic efficacy of IP10-EGFRvIIIscFv fusion protein on glioblastoma; however, brain targeting delivery systems can transport drugs directly to the brain to treat glioblastoma. Brain targeting delivery systems mediated by receptors, such as nanoparticles (NPs), are among the most effective ones^27^. Angiopep-2 modified nanoparticles facilitate IP10-EGFRvIIIscFv fusion protein to pass the BBB and bind to the EGFRvIII antigen on glioblastoma cells.

In the current study, we established a new cascade targeting delivery system, which uses both angiopep-2 and IP10-EGFRvIIIscFv fusion protein modified nanoparticles (AINPs) to deliver the EGFRvIIIscFv fusion protein into brain malignant glioblastoma cells with accuracy. Both *in vitro* and *in vivo* experiments were performed, which confirmed the strong ability of AINPs to traverse BBB, and the therapeutic effect of AINPs combined with CTL on glioblastoma *in vivo* was encouraging.

## Results

### Characterization of AINPs

**Figure 1:**
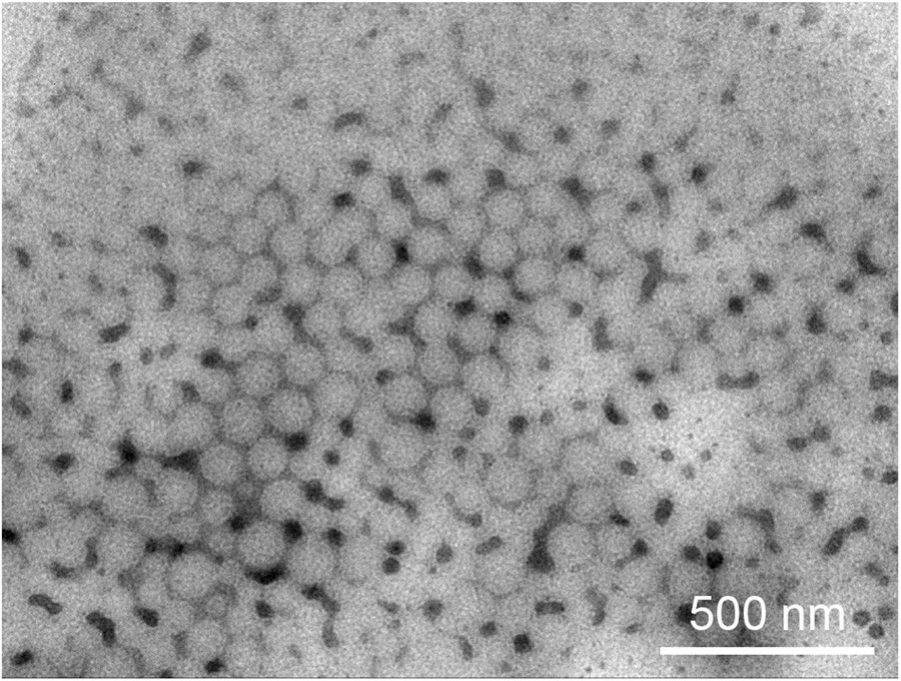
Transmission electron micrograph of AINPs negatively stained with phosphotungstic acid solution (bar: 500 mm).

Morphology of different NPs, in presence or absence of IP10-EGFRvIIIscFv and/or angiopep-2, NPs, (Angiopep-2 modified nanoparticles)ANPs, INPs and AINPs negatively stained with 2% sodium phosphotungstate solution, were observed under TEM. NPs were found to be of uniform size and well-formed spherical shape with compact structure (Fig. 1). The particle size and zeta potential of NPs were further detected with DLS) (Table 1). Average size of NPs was 121.8 ± 1.1 nm, Zeta potential was −26.49 ± 3.03 mV. After conjugation with IP10-EGFRvIIIscFv and/or angiopep-2, particle sizes showed a little bit increase for ANP, INP and AINP (125.8 ± 0.3 nm, 134.7 ± 0.6 nm and 143.6 ± 0.7 nm, respectively).

**Table 1:** Particle size and Zeta potential of various nanoparticles.

Nanoparticles	Average particle size	Zeta potential (mV)(mean ± SD, nm)
NPs	121.8 ± 1.1 nm	−26.49 ± 3.03 mV
ANPs	125.8 ± 0.3 nm	−26.09 ± 7.0 mV
INPs	134.7 ± 0.6 nm	−24.71 ± 2.67 mV
AINPs	143.6 ± 0.7 nm	−25.61 ± 3.53 mV

Zeta potential of each nanoparticle was determined in 0.1 M PBS.

### Uptake of NPs by bEnd.3 cells *in vitro*

**Figure 2:**
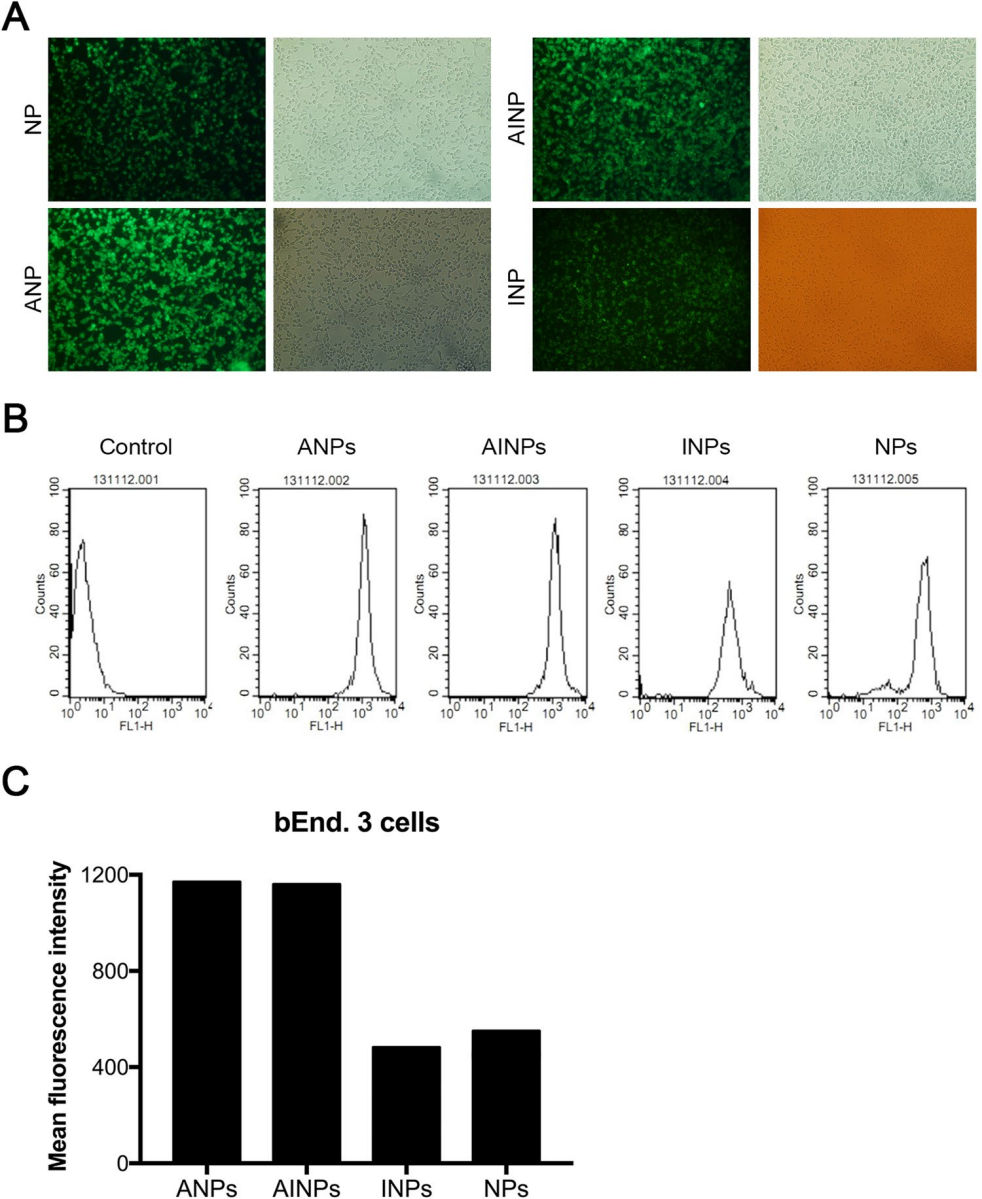
The bEnd.3 cells pre-incubated with NPs, ANPs, INPs or AINPs, respectively, were observed under a fluorescence microscope (**A**) for qualitative assessment and (**B**) for assessment of *in vitro* uptake of coumarin-6-loaded NP, INP, AINP and ANP by bEnd.3 cells. (**C**) Quantitative assessment of uptake (**B**), and statistical histogram. The average fluorescence quantification values of ANPs, AINPs, INPs, and NPs in bEnd.3 cells containing coumarin-6 were 1165.32, 1157.03, 489.31, and 559.01, respectively. The fluorescence intensity of ANPs and AINPs nanoparticles with Angiopep-2 targeting head was stronger than that of NPs and INPs without Angiopep-2 targeting head, which indicates effective uptake of our nanoparticles with angiopep-2 targeting heads by mouse brain capillary endothelial cells.

In order to determine ability of bEnd.3 cells to take up different NPs in presence or absence of IP10-EGFRvIIIscFv fusion protein and/or angiopep-2, bEnd.3 cells were pre-incubated with NPs, ANPs, INPs or AINPs, respectively, at 37 °C for 0.5 h. The bEnd.3 cells were encapsulated with fluorescein mounting solution after fixing with 4% paraformaldehyde, and observed with fluorescence microscopy. Figure 2A shows that the average fluorescence quantitative values were 1165.32, 1157.03, 489.31, and 559.01 for coumarin-6-loaded NP, INP, AINP, and ANP, respectively. Figure 2B shows the quantitative analysis based on flow cytometry. These results indicate that the cell fluorescence intensity of ANPs and AINPs nanoparticles was stronger than that of NPs and INPs.

The fluorescence intensity of INPs was similar to that of NPs, while the fluorescence intensity of ANPs and AINPs was higher than that of NPs and INPs. These findings indicate effective uptake of nanoparticles with Angiopep-2 by the bEnd.3 cells (Fig. 2C).

### Fluorescence distribution and intensity of different NPs *in vivo*

**Figure 3:**
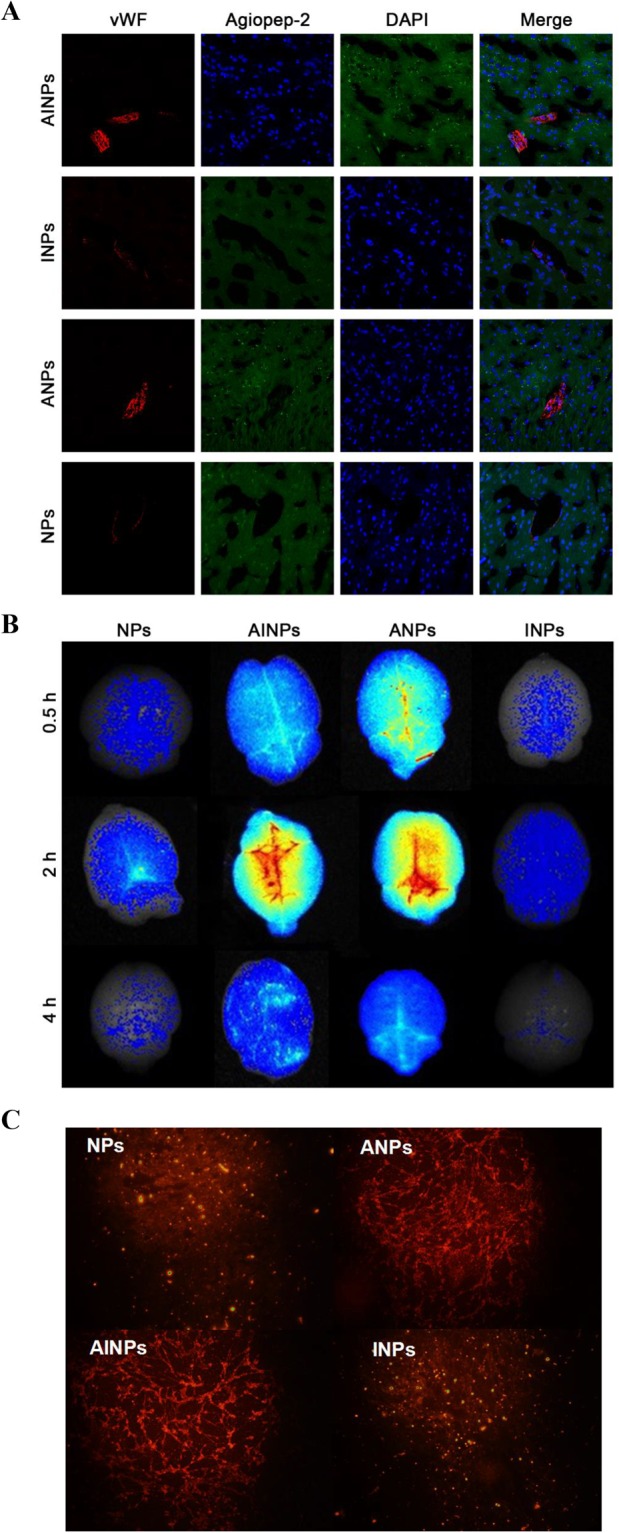
Determination of distribution and intensity of NP, ANP, INP and AINP in the rat brain tissues by fluorescence imaging after treatment with NP, ANP, INP and AINP. (**A**) Fluorescence images, endothelial cells are stained in blue, the neuroglial cells are stained in red and green color represents the NPs; (**B**) *ex vivo* fluorescence imaging of brain separated from BALB/c nude mice 0.5 h, 2 h and 4 h after intravenous administration of NP, ANP, INP and AINP. (**C**) RhoB-Labeled NPs, ANPs, AINPs, and INPs ingested by BCECs.

To investigate distribution of NPs with or without IP10-EGFRvIIIscFv and/or angiopep-2 *in vivo*, 100 mg/kg of coumarin-6-containing NPs, ANPs, INPs or AINPs was injected into each mouse via tail vein, respectively. Thirty minutes post-injection, mice were perfused with 4% paraformaldehyde for 30 min. Brain tissues of mice were removed and 5-μm thick sections prepared. Fluorescence intensity of brain tissues was observed under confocal microscope after double staining by von Wilebrand factor (VWF)^28,29^ and DAPI. Distribution of different NPs in mouse brain was reflected by the fluorescence intensity. Particle numbers with Angiopep-2 (AINPs and ANPs) in brain tissues were significantly higher than particles without Angiopep-2 (INPs and NPs), which was evidenced by the fluorescence distributions and intensities of different NPs *in vivo* (Fig. 3A). This indicated that Angiopep-2 effectively facilitated passage of NPs across BBB *in vivo*.

### ***Ex vivo*** fluorescence imaging of brain for further determination of NP distribution and intensity

For fluorescence imaging of brains with different NPs with or without the IP10-EGFRvIIIscFv fusion protein and/or angiopep-2, complete brain specimens were separated from BALB/c nude mice, which were injected with 100 mg/kg DiR containing NPR, AINPs, ANPs or INPs via the tail vein, and observed with a living Maestro 2 *in vivo* imaging system. Different types of particles showed different fluorescence intensity in the whole brain tissues. The fluorescence intensity of AINPs and ANPs in the brain tissues of nude mice was obviously stronger than that of NPs or INPs; the difference was most significant at 2 h post-injection and gradually diminished at 4 h (Fig. 3B), which was further evidenced in the uptake of RhoB-Labeled NPs, ANPs, AINPs and INPs by BCECs. These results further confirmed that angiopep-2 facilitated the passage of NPs across the BBB.

### Determination of the binding ability of specific antibody of EGFRvIIIscFv in AINPs to the receptor of EGFRvIII on the surface of glioblastoma cells

Glioblastoma cells U87-EGF12RvIII with EGFP fluorescence tag and stable expression of EGFRvIII could be specifically recognized by EGFRvIII monoclonal antibody.

To identify the binding ability of the specific antibody against EGFRvIII to the receptor of EGFRvIIIscFv in AINPs on the surface of glioblastoma cells, the NPs not modified by IP10-EGFRvIIIscFv (NPs and ANPs) were used as negative controls, while the NPs modified by IP10-EGFRvIIIscFv (INPs) were used as positive controls.

The U87-EGFRvIII cell line was U87 cell line transfected with EGFP lentivirus, which emits green light under fluorescent microscope and confocal laser scanning microscope. The U87-EGFRvIII cell line has stable expression of EGFRvIII, and is specifically recognized by EGFRvIII monoclonal antibody.

**Figure 4:**
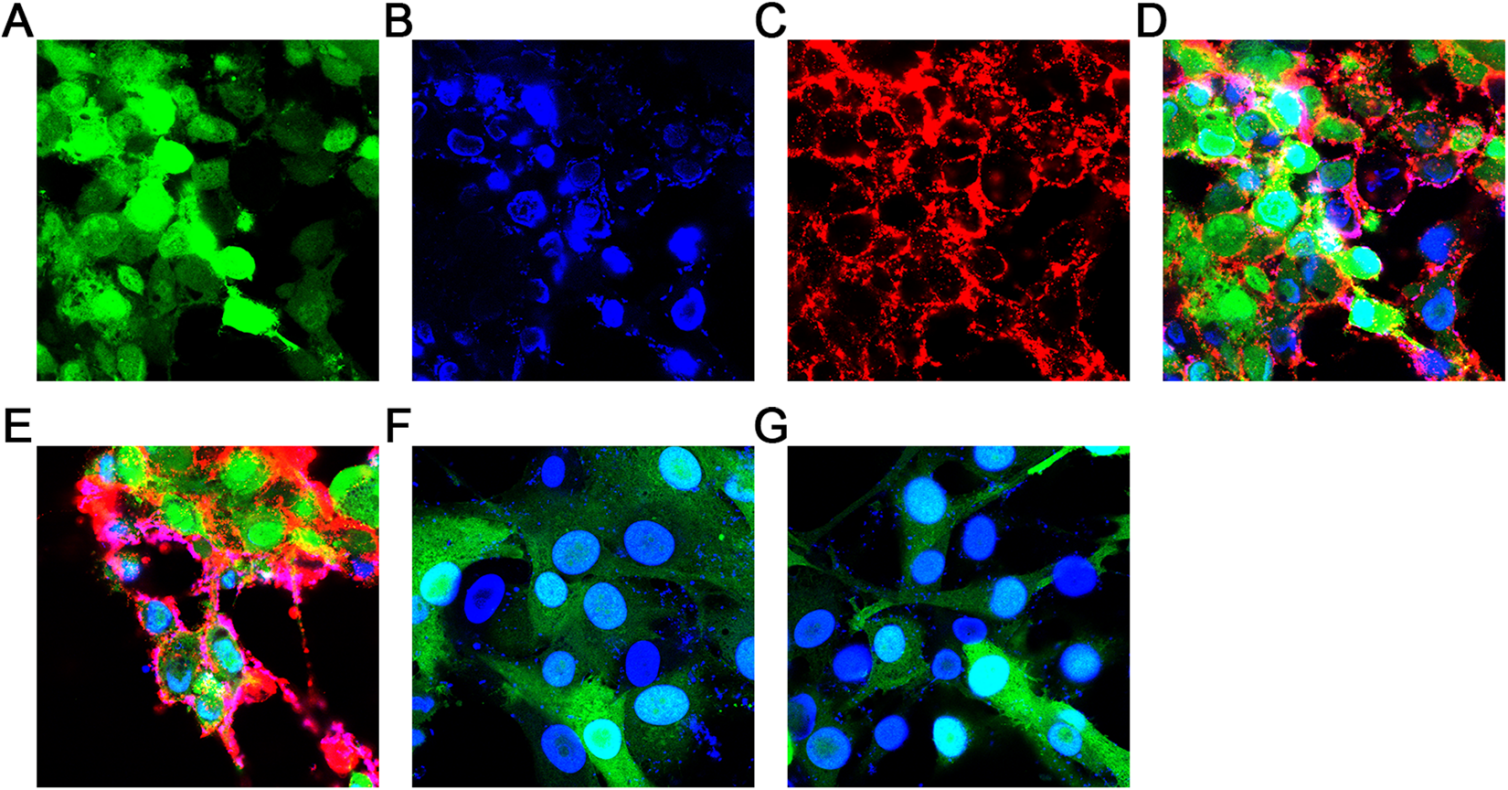
Binding ability of AINPs to glioblastoma cells (U87MG) determined by confocal microscopy. The surface of AINPs and INPs used in this study were modified by the fusion protein IP10-EGFRvIII (specific binding to EGFRvIII) and a His6-Tag. The Dylight650 labeled anti-His6mAb was used and showed red fluorescence under fluorescence microscopy and laser confocal microscopy. (**A**–**D**) Represent EGFP, DAPI, Dy650light and merged images of AINPs. (**E**–**G**) Are the merged images of INPs, ANPs and NPs.

The AINPs and INPs prepared on our surface have been modified with the fusion protein IP10-EGFRvIIIscFv. The EGFRVIII scFv part specifically binds to EGFRvIII, and the fusion protein also has the His6-Tag tag. We chose the Dylight650-labeled Anti-His6 mAb to specifically bind to it. Fluorescence microscope or laser confocal microscope showed red light. We prepared AINPs and INPs modified with the fusion protein IP10-EGFRvIIIscFv, and the EGFRVIII scFv part specifically binds to EGFRvIII. Furthermore, the fusion protein also has a His6-Tag tag. After binding of Dylight650-labeled Anti-His6 mAb to the His6-tag, it emits red fluorescence on fluorescence microscopy or laser confocal microscopy. To identify the ability of AINPs to bind to EGFRvIII in tumor cells, we used the non-fusion protein IP10-EGFRvIIIscFv modified NPs and ANPs as the negative control group and INPs as the positive control group. The results are shown in Fig. 4 (A, B, C and D show EGFP, DAPI, Dy650light and merger of the AINPs group, respectively; E, F, and G are the merged images of the INPs group, the ANPs group, and the NPs group, respectively). Red fluorescence was observed only in the AINPs and INPs groups, which indicates that NPs modified IP10-EGFRvIII fusion proteins effectively targeted the glioblastoma cells (Fig. 4).

### Effects of AINPs on CD8^+^ T lymphocyte migration

**Figure 5:**
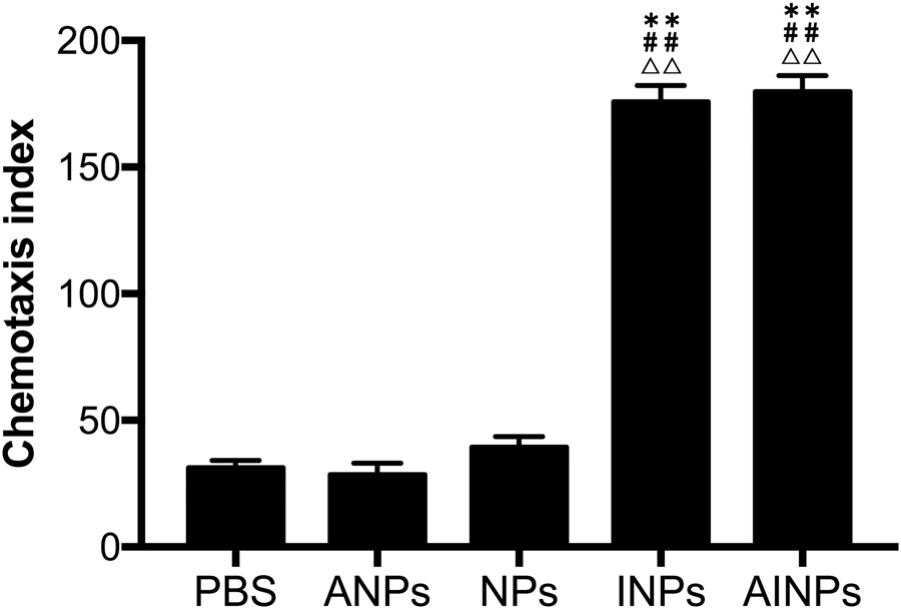
Chemotaxis index of activated CD8^+^ T lymphocytes in response to different type of NPs. **p < 0.01 versus the PBS group. ^##^p < 0.01 versus the ANPs. **p < 0.01 versus the NPs.

To determine whether the IP10-EGFRvIIIscFv fusion proteins in the AINPs had the capacity to facilitate transendothelial chemotaxis required for recruitment of CTL to the target glioblastoma cells, we prepared activated CD8^+^ T cells *in vitro*. The chemotactic effects of NPs, ANPs, INPs, AINPs at 250 μg/mL or PBS only were determined with transwell migration assay. The chemotactic indices of the AINPs and INPs on the activated CD8^+^ T cells were significantly higher (*p* < 0.0001) than that of PBS; however, the chemotactic indices of NPs and ANPs on the activated CD8^+^ T cells showed no significant difference versus PBS (*p* > 0.05) (Fig. 5). The results indicated that the IP10-EGFRvIIIscFv fusion protein contained in the prepared AINPs maintained immunoreactivity and showed good chemotactic activation of CTL.

### Anti-glioblastoma effect of AINPs *in vivo*

**Figure 6:**
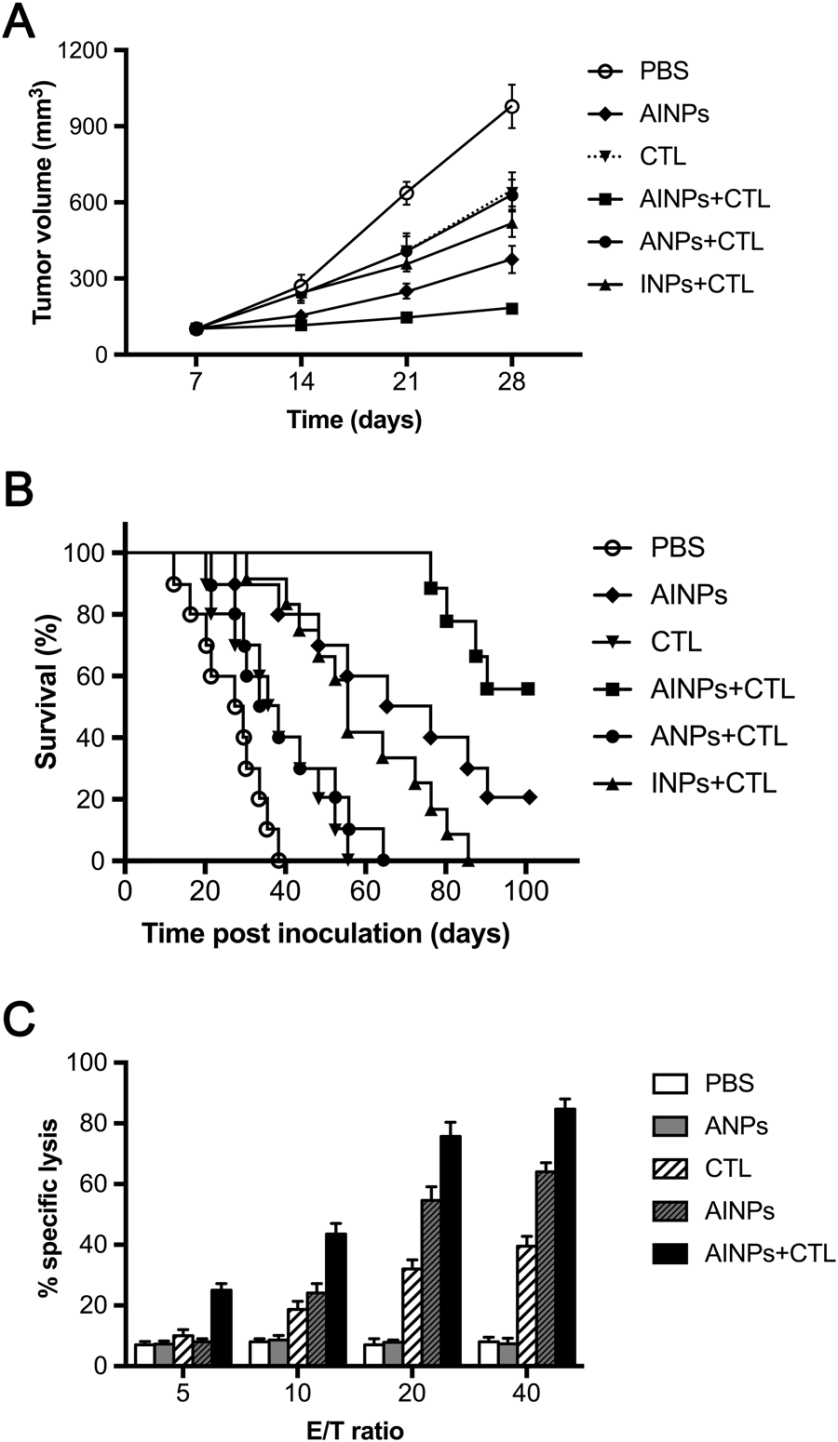
Anti-glioblastoma effect of AINPs *in vivo*. BABL/C/Nude mice with intracranial inoculation of U87-EGFRvIII cells were randomly divided into six groups (n = 10 each). On the 7^th^, 14^th^, and 21^st^ day, the following reagents were injected into the tail vein of mice in the respective groups. (1) AINPs + CTL; (2) 2INPs + CTL; (3) CTL; (4) ANPs + CTL; (5) AINPs; (6) PBS. Tumor volume was calculated based on the formula: (maximum diameter) × (vertical diameter)^2^ × 0.5 (**A**); the number of mice that died was recorded and the survival curves made according to the survival rates of mice during the period of 100 days after inoculation (**B**); interaction of U87MG-EGFRvIII with CTL at different ratios (**C**), brain-infiltrating leukocytes (BILs) were isolated from the AINPs + CTL, AINPs, CTL, ANPs and PBS-treated mice post-inoculation and co-cultured with Cr^51^-labeled U87-EGFRvIII cells *in vitro*. Activated lymphocytes were then seeded into a 96-well microtiter plate with Cr51-labeled U87MG-EGFRvIII cells at a ratio of 40:1, 20:1, 10:1 or 5:1 and incubated for 6 h at room temperature. The radioactivity level per minute was calculated.

We further evaluated anti-glioblastoma effect of AINPs on life span *in vivo* (Fig. 6). BALB/c nude mice were inoculated intracranially with U87MG-EGFRvIII cells to establish a xenograft glioblastoma model. The mice with xenograft glioblastoma were then randomly assigned to AINPs + CTL, INPs + CTL, CTL, ANPs + CTL, AINPs or PBS groups, and intravenously injected with AINPs + CTL, INPs + CTL, CTL, ANPs + CTL, AINPs or PBS at days 7, 14 and 21 post-inoculation, respectively. Mice were sacrificed by cervical dislocation at days 7, 14 and 21 post-inoculation and brain tissues removed. Tumor diameter was measured with vernier caliper. Tumor volume was calculated according to formula:

$$({\rm{largest}}\,{\rm{diameter}})\times {({\rm{perpendicular}}{\rm{diameter}})}^{2}\times \mathrm{0.5.}$$

Tumor volume in AINPs + CTL group was significantly lower in INPs + CTL, CTL, ANPs + CTL, AINPs and PBS groups (*p* < 0.05) (Fig. 6). Mice in INPs + CTL, CTL, ANPs + CTL and PBS groups started to die at the 80^th^ day, 55^th^ day, 64^th^ day and 40^th^ day post-inoculation of U87-EGFRvIII cells, respectively. Two and five mice in AINPs and AINPs + CTL groups, respectively, were still alive at 100^th^ day post-inoculation (Fig. 6).

Cytotoxicity of activated tumor specific CTLs against glioblastoma *in vivo* was further evaluated. Brain-infiltrating leukocytes (BILs) were isolated from the AINPs + CTL, AINPs, CTL, ANPs and PBS-treated mice post-inoculation and co-cultured with Cr^51^-labeled U87-EGFRvIII cells *in vitro*. Activated lymphocytes were then seeded into a 96-well microtiter plate with Cr51-labeled U87MG-EGFRvIII cells at a ratio of 40:1, 20:1, 10:1 or 5:1 and incubated for 6 h at room temperature. The radioactivity value per minute was calculated. Radioactivity of AINPs + CTL, AINPs and CTL groups were highest at 40:1 ratio, while that of PBS and ANPs groups showed no difference with change in the ratio (p > 0.05) (Fig. 6C). The radioactivity of AINPs + CTL and AINPs groups showed significant difference at different ratios (p < 0.05). The radioactivity of AINPs + CTL group showed most significant difference compared with other groups at each ratio (p < 0.01). These results suggested that BILs in AINPs + CTL treated mice showed strong anti-glioblastoma activity.

## Discussion

Advances made in lymphocyte co-culture technology allow generation of specific toxic lymphocytes, such as CTL after co-culture of T-lymphocytes with dendritic cells (the antigen-presenting cells). Recently developed ability to specifically amplify glioblastoma-specific lymphocytes *in vitro* represents a major breakthrough in this field. Tumor-specific CTLs were re-transfused into human body to facilitate binding of effector cells to target cells and exert cytotoxic effect on tumor cells^30^.

Tumor microenvironment refers to tumor cells, non-tumor cells, collagen and soluble secretions and other extracellular matrix^31^. The immune escape function of tumor cells is related to inhibitory immune cell activity in tumor microenvironment, while tolerogenic DCs, regulatory T lymphocytes (T_*reg*_) and tumor associated macrophage are the most common inhibitory immune cells.

T_*reg*_ cells can infiltrate many malignancies in large numbers, and infiltrated T_reg_ cell number is negatively related with tumor development and prognosis. Therefore, inhibiting T_*reg*_ cell function can effectively restore the anti-tumor immune function of the human body and promote CTLs to effectively play the anti-tumor role^32,33^.

However, due to limited CTL numbers in human body and restriction of dependent APC activation, including immune escape mechanisms and inhibitory effect of T_reg_ cells, limits CTL numbers directed to glioblastoma cells, therefore, strategies to enrich CTLs in glioblastoma cell locations may improve therapeutic efficacy.

CXCR3 is mainly distributed in cytotoxic TH_1_ effector T, NK, activated B and CD8^+^ T cells. It mediates monocyte chemotactic movement and immune surveillance^34^. As a chemokine ligand, binding of CXCL10 (IP10) to CXCR3 can induce cell proliferation and chemotaxis.

Cell signaling induced by EGFRvIII potentiates anti-apoptotic ability of tumor cells. In addition to targeting of glioblastoma cells, EGFRvIIIscFv can also inhibit the invasion and proliferation of tumor cells and promote their apoptosis through signal transduction pathway.

Therefore, IP10-EGFRvIIIscFv fusion protein may specifically bind to EGFRvIII-positive glioblastoma cells and result in production of high IP10 concentration in vicinity of glioblastoma cells. In addition to its anti-angiogenic effect, IP10 can enrich adopt CTLs to glioblastoma cells, and eventually form the sites to eliminate glioblastoma cells around EGFRvIII-positive cells.

The cellular uptake results suggested the angiopep-2 only targeted the endothelial cells while IP10-EGFRvIIIscFv fusion proteins only targeted the glioblastoma cells; further, IP10 could facilitate transendothelial chemotaxis required for recruitment of CTLs to the target glioblastoma cells. Therefore, we further conjugated the AINPs, to provide a cascade targeting delivery system. This is because individually these could not enter both endothelial and glioblastoma cells and cross the BBB into the brain parenchyma. At the same time, we continued to investigate whether AINPs could effectively facilitate chemotaxis of CD8^+^ T lymphocytes by IP10 and the EGFRvIIIscFv to targete the EGFRvIII positive glioblastoma cells. Finally, we studied the overall therapeutic effect of AINPs in the mouse model of *in situ* glioblastoma.

Transwell migration assay showed that the chemotaxis of CD8^+^ T lymphocytes in response to IP10-EGFRvIIIscFv fusion protein modified AINPs and INPs was significantly greater than that induced by EGFRvIIIscFv-modified ANPs and NPs (p < 0.0001); however, there was no significant difference in this respect between NPs and the ANPs (p > 0.05), which indicates that the AINPs showed excellent chemotaxis of activated CD8^+^ T Lymphocyte.

In the antigen-antibody binding test, only the AINPs and INPs showed red light fluorescence, which indicated that our nanoparticle-modified IP10-EGFRvIII fusion proteins showed active targeting of the glioblastoma cells.

Combined treatment of AINPs with CTL of the glioblastoma *in vivo* showed that mice in the INPs + CTL, CTL, ANPs + CTL and PBS group started to die by the 80^th^, 55^th^, 64^th^ or 40^th^ day post-inoculation, respectively, of U87-EGFRvIII cells, while two and five mice in AINPs and AINPs + CTL groups were still alive at 100^th^ day post-inoculation, respectively.

Radioimmunoassay cytotoxicity assay showed that AINPs + CTL and AINPs had different cytotoxic effects on glioblastoma cells at different ratios of the effector cells and the target cells (p < 0.05). In addition, the differences in the cytotoxic effects on glioblastoma cells were more significant between AINPs + CTL group and the other groups (p < 0.01).

These results suggested strong cytotoxic effect of the infiltrated lymphocytes against glioblastoma cells in the AINPs + CTL group. The therapeutic effect of AINPs was related to their inhibitory effect on tumor angiogenesis and their regulatory effect on apoptosis by IP10-EGFRvIIIscFV; however, the therapeutic effect of AINPs + CTL combined treatment was better than that of AINPs alone.

In the present study, we used a new cascade targeting strategy with angiopep-2 and IP10-EGFRvIIIscFv fusion proteins, and assessed the efficacy of the strategy for targeting of brain glioblastoma cells and recruitment of activated CD8^+^ T lymphocytes to the location of glioblastoma cells. We founded that AINPs interacted specifically with bEnd.3 cells. In addition, we demonstrated that AINPs accumulated in brain tissue after intravenous injection and recruited the activated CD8^+^ T lymphocytes to the location of glioblastoma cells. These results suggested that AINPs may be useful for brain-targeted delivery and for recruitment of activated CD8^+^ T lymphocytes to the glioblastoma cells.

## Materials and Methods

### Materials and Reagents

Angiopep-2 (TFFYGGSRGKRNNFKTEEYC) was synthesized by Hangzhou middle peptide biochemical Co., Ltd (Hangzhou, China); poly (lactic-co-glycolic acid) (PLGA), PLGA-PEG-Mal(lactic-co-glycolic acid-polyethylene glycol maleamide) were provided by Professor Zhiping Zhang; IP10-EGFRvIIIscFv recombinant gene was constructed by Shanghai Genechem Co., Ltd. (Shanghai, China); IP10-EGFRvIIIscFv fusion protein was purified by GenScript (Shanghai Co., Ltd., Shanghai, China); 6-coumarin, N-(3-dimethylaminopropyl)-N0-ethylcarbodiimide hydrochloride (EDC) and N-hydroxysuccinimide (NHS), DAPI reagent, 1,10-Dioctadecyl-3,3,30,30-tetramethylindotricarbocyanine Iodide (DiR), TPGS(d-α-Tocopheryl polyethylene glycol 1000 succinate) were obtained from Sigma-Aldrich Co., LLC. (St. Louis, USA). Fetal bovine serum (FBS) was purchased from Gibco (CA). All the other chemicals were analytical reagent grades, purchased from Sinopharm Chemical Reagent (China).

### Cell lines, cell cultures and treatments

Endothelial cell line bEnd.3 was purchased from GuangZhou Jennio Biotech Co.,Ltd.; U87MG-EGFRvIII cell line, U87MG (a human primary glioblastoma cell line) stablely expressed EGFRvIII was previously established in our laboratory. bEnd.3 cells were cultured in DMEM with 100 U/mL penicillin, 100 mg/mL streptomycin and 10% FBS. U87-MGvIII cells were cultured in DMEM (GIBCO, Invitrogen) with 2 mM L-glutamine, 100 U/mL penicillin, 100 mg/mL streptomycin and 10% FBS. This study was approved by The Ethics Committee of Tongji Medical College, Huazhong University of Science and Technology. All experiments were performed in accordance with relevant guidelines and regulations.

### Preparation of nanoparticles

A total of 16 mg PLGA and 4 mg PLGA-PEG-Mal were completely dissolved in 2 mL acetone solution, followed by slowly added 0.1% TPGS aqueous solution drops over 3–5 minutes. The mixture was stirred overnight on a magnetic stirring apparatus to let the organic solvent being fully evaporated, then centrifuged at 1500 rpm for 10 min, at 12000 rpm for 10 min × 2 times. The pellet was re-suspended in 2 mL of PBS (pH 7.4), which was uses as NPs (without any of angiopep-2 or IP10-EGFRvIIIscFv).

For IP10-EGFRvIIIscFv conjugation, the carboxyl unit of NPs was activated by EDC and NHS in MES buffer (pH 6.0) for 0.5 h; MES buffer was then replaced by pH 7.4 PBS using Hitrap^TM^ desalting column. A 0.4 mg of IP10-EGFRvIIIscFv fusion protein in 2 mL of PBS (pH 7.4) was added into the NPs suspension prepared above and stirred for 8 h in dark. The conjugated IP10-EGFRvIIIscFv fusion protein was applied to a sepharose PD-10 column to remove unconjugated IP10-EGFRvIIIscFv, and the purified product was uses as INPs (NPS with IP10-EGFRvIIIscFv).

For angiopep-2 conjugation (PLGA-PEG-MAL/Angiopep-2 1/1, mol/mol), 500 μg angiopep-2 was added to the prepared suspension of NPs and stirred for 6 h in dark. The mixture was centrifuged at 1500 rpm for 10 min, at 12000 rpm for 10 min × 2 times. The pellet was re-suspended in 2 mL of PBS (pH 7.4) and purified on a PD-10 column to remove the free Angiopep-2, and the purified product was uses as ANPs (NPS with angiopep-2).

IP10-EGFRvIIIscFv fusion protein modified NPs (INPs) were prepared as above without adding angiopep-2.

Coumarin-6-loaded AINPs were prepared as above with 50 μg/mL coumarin-6 and dissolved in 2 mL of acetone solution when NPs were prepared.

DiR is a lipophilic, near-infrared fluorescent cyanine dye that can be used as a tracer. DiR-loaded AINPs were prepared as described above; in addition 50 μg/mL DiR (instead of PLGA and PLGA-PEG-Mal) was dissolved in 2 mL of acetone solution during preparing NPs.

### Characterization of angiopep-2 modified nanoparticles (AINPs) detected by transmission electron microscope

A total of 10 µl nanoparticle suspension on the copper grid was air dried overnight, and negatively stained with 2% sodium phosphotungstate solution. The morphology was examined with transmission electron microscope (TEM; Hitachi, Japan). The size and Zeta potential of nanoparticles were detected by dynamic light scattering (DLS) analysis using a Zeta Potential & Particle Size Analyzer (Brookhaven Instruments, Holtsville, USA).

### *In vitro* cell uptake detected by microscopy

The bEnd.3 cells were seeded with 1 × 10^4^ cells/mL on coverslips pre-loaded in 24-well plates for 24 h, and then pre-incubated with Hank’s Balanced Salt Solution (HBSS) for 10 min. The HBSS solution was removed and PBS solution (250 μg/mL) with coumarin-6-loaded NP, ANPs, INPs or AINPs was added and incubated at 37 °C for 0.5 h. PBS solution with coumarin-6-loaded NP, ANPs, INPs and AINPs was discarded. Coverslips were removed from 24-well plates after washing and placed in a petri dish containing 4% paraformaldehyde for 30 minutes at room temperature, rinsed again with PBS, encapsulated with fluorescein mounting solution, observed with phase contrast microscopy (CKX41, Olympus, Japan) and fluorescence microscopy (BX53, Olympus, Japan).

### Quantitative analysis of NPs uptake by bEnd.3 cells using flow cytometer

To quantitatively analyze the ability of bEnd.3 cells in taking up different NPs in presence or absence of IP10-EGFRvIIIscFv fusion protein and/or angiopep-2, bEnd.3 cells pre-incubated with NPs, ANPs, INPs or AINPs, respectively, and the fluorescence intensity was detected by flow cytometry (BD Biosciences, USA) analysis with the flow speed of 10 ul/min, excitation wavelength of 466 nm and emission wavelength of 504 nm, total cell number of 1000000.

For quantitative analysis, bEnd.3 cells were seeded to T25 cell culture flasks and treated as described above. Fluorescence intensity.

### *In vitro* antigen binding assay

U87MG-EGFRvIII and U87MG cells with stable expressing EGFRvIII and EGFP fluorescence labeling were seeded on cover slips pre-coated with polylysine in 24-well plate. Cells were then incubated in DMEM medium supplemented with high glucose, 100 U/mL penicillin, 100 mg/mL streptomycin and 10% FBS for 2–3 days until the cell density reached more than 80%. The cell culture medium was discarded. The cells were washed twice with PBS and fixed with 4% paraformaldehyde for 20 min. Cells were washed 3 times with PBS containing 2% BSA (PBA) and treated with 0.1% TritonX-100 for 20 min, washed 3 times with PBS and blocked with 3% BSA for 30 min, then added NPs, ANPs, INPS or AINPs (10 μg/mL) respectively, incubated overnight at 4 °C, washed 3 times with PBA, and incubated with Dylight 650-labeled Anti-His6 monoclonal antibody (1 μg/mL) for 1–2 h and with DAPI reagent for 5 to 10 minutes. The slides were sealed with fluorescent mounting reagent. Confocal microscopy (FV1000, Olympus, Japan) was used to examine the fluorescence.

### *In vitro* transwell migration assay

Transwell with 5 μm aperture were used. Cells were re-suspended in PBS with 1% BSA and titrated to a concentration of 10^6^/mL. The upper chamber of the transwell was filled with 200 μL cell suspension and the lower chamber of the transwell was filled with 500 μL PBS solution containing NPs, ANPs, INPs or AINPs (250 μg/mL) or only PBS. The underlayer of the cell permeable membrane of the transwell was evenly smeared with 5 mg/mL fibronectin and incubated at 37 °C for 4 h to block. The cell permeable membrane was washed with PBS. Cells on the upper layer of the cell permeable membrane which did not migrate across the membrane were gently wiped off with cotton bud. The cells that had migrated to the lower layer of the cell permeable membrane were fixed with 4% paraformaldehyde for 15 min. After drying, the cells were stained with 500 μL 0.5% crystal violet dye for 1 hour. The number of migrated cells was observed under a microscope after decoloration. Migrated cell number was determined by the total number in 5 high power fields. The chemotactic index (CI) was the CD8^+^ lymphocyte ratio between the number migrating to the nanoparticle suspension and the number migrating to PBS.

### *In vivo* fluorescent assay

SPF grade BALB/c nude mice (age: 4–5 weeks) were maintained under standard housing conditions. All animal experiments were carried out in accordance with guidelines evaluated and approved by the ethics committee at the Tongji Medical College, Huazhong University of Science and Technology, and conducted in the SPF level laboratory of the experimental animal center.

Twelve 6–8 week-old female BALB/c nude mice were randomly divided into 4 groups: NPs, ANPs, INPs and AINPs groups. Each mouse was injected with 100 mg/kg of coumarin-6-containing NPs, ANPs, INPs or AINPs via the tail vein. Thirty minutes post-injection, mice were anesthetized with ether, perfused with normal saline for 20 min, and then perfused with 4% paraformaldehyde for 30 min. The mice were sacrificed by cervical dislocation and the brain tissue was removed. The brain tissues were coronally cut at 1 cm after the optic chiasm and washed 10 times with PBS, and then placed in 30%, 20% and 10% sucrose solutions, in turn, overnight. The brain tissues were wrapped in a foil and stored at −80 °C in a refrigerator for future use. The brain tissues were then embedded with Tissue Tek OCT compound and frozen at −80 °C, and were successively cut into 5-μm thick slices with the cryotome Cryostat (Leica, CM 1950, Germany). Endothelial cells of the frozen slices were stained by VWF and DAPI reagent was added for 5 to 10 minutes to stain the nucleus and sealed with fluorescent mounting solution. The fluorescence intensity of brain tissues was observed under a confocal microscope (OLS4500, Olympus, Japan).

### *In vitro* imaging

Twelve SPF BALB/c nude mice were randomly divided into NPs, AINPs, ANPs and INPs groups. Each mouse was injected with 100 mg/kg DiR contained NPR, AINPs, ANPs or INPs via tail vein. At 0.5 h, 2 h and 4 h post-injection, one mouse from each group was randomly selected, anesthetized and sacrificed by cervical dislocation. The complete brain specimens were tiled on partition and observed with living Maestro 2 *in vivo* imaging system (Caliper Life Sciences, PerkinElmer Company, Hopkinton, MA).

### Tumor challenge

A total of 48 BALB/c nude mice were anesthetized and inoculated intracranially with 2 × 10^4^ U87MG-EGFRvIII cells in 2 μL PBS. Cells were injected stereotactically through an entry site at the bregma 2 mm to right of sagittal suture and 3 mm below the surface of skull of mice utilizing a Reword stereotactic frame (Reword Instrument, China). Mice were randomly divided into 6 groups (n = 8). NPs + U87-EGFRvIII-specific DCs induce CTL, ANPs + CTL, INPs + CTL, AINPs + CTL and PBS were administrated, respectively, by tail vein injection at 7, 14 and 21 days after tumor cells were inoculated. The changes in mental status, behavior and diet of mice were observed. Diameters of tumors were measured and tumor volumes were calculated: tumor volume = d1 × (d2)^2^ × 0.5 (d1, largest diameter; d2, perpendicular diameter).

### Cytotoxicity assay

The cytotoxicity of CTL against glioblastoma U87MG-EGFRvIII cells was determined by 51Cr-release assay with minor alteration. In brief, brain-infiltrating leukocytes (BILs) were isolated from the AINPs and CTL, AINPs, CTL, ANPs or PBS-treated mice on day 14 post-inoculation and stimulated with U87MG-EGFRvIII cells in RPMI1640 with 10% FBS for 5 days. The activated cells were then used as effector cells. 100 μL Cr51-labeled U87MG-EGFRvIII cells were seeded in a 96 well plate, and activated lymphocytes were then added to make the ratio of activated lymphocytes: Cr51-labeled U87MG-EGFRvIII cells as 40:1, 20:1, 10:1 or 5:1. The same volume of complete medium without activated lymphocytes was added in the blank control group; the same volume of 1% NP-40 lysate was added in the positive control group. Each group was conducted with triplication. Cells were then cultured in a 37 °C incubator for 5 h after centrifugation at 100 rpm for 5 min. Then the 96 well plate with cells was centrifuged at 600 g for 5 min, and 100 μL of supernatant was used to determine the counts per minute (cpm) using the liquid scintillation counter. Cytotoxicity rate was calculated by the formula:

$$\begin{array}{c}{\rm{Cytotoxicity}}\,( \% )=\{[{\rm{CPM}}({\rm{experimentalgroup}})])-{\rm{CPM}}({\rm{spontaneous}})/\\ \,\,\,\,\,\,\,\,{\rm{CPM}}({\rm{maximum}})-{\rm{CP}}({\rm{spontaneous}})]\}\times {\rm{100}}{\rm{.}}\end{array}$$
