# Author Correction: Angiopep-2/IP10-EGFRvIIIscFv modified nanoparticles and CTL synergistically inhibit malignant glioblastoma

**DOI:** 10.1038/s41598-019-40291-5

**Published:** 2019-03-12

**Authors:** Xuan Wang, Zhiyong Xiong, Zhen Liu, Xing Huang, Xiaobing Jiang

**Affiliations:** Department of Neurosurgery, Wuhan Union Hospital, Tongji Medical College, Huazhong University of Science and Technology, Wuhan, 430022 China

Correction to: *Scientific Reports* 10.1038/s41598-018-30072-x, published online 27 August 2018

The original version of this Article contained an error in the Affiliation, which was incorrectly given as ‘Department of Neurosurgery, Tongji Medical College, Huazhong University of Science and Technology, Wuhan, 430022, China’. The correct affiliation is listed below:

Department of Neurosurgery, Wuhan Union Hospital, Tongji Medical College, Huazhong University of Science and Technology, Wuhan, 430022, China

This error has now been corrected in the HTML and PDF versions of the Article.

